# Ventilatory support and mechanical properties of the fibrotic lung acting as a “squishy ball”

**DOI:** 10.1186/s13613-020-0632-6

**Published:** 2020-02-04

**Authors:** Alessandro Marchioni, Roberto Tonelli, Giulio Rossi, Paolo Spagnolo, Fabrizio Luppi, Stefania Cerri, Elisabetta Cocconcelli, Maria Rosaria Pellegrino, Riccardo Fantini, Luca Tabbì, Ivana Castaniere, Lorenzo Ball, Manu L. N. G. Malbrain, Paolo Pelosi, Enrico Clini

**Affiliations:** 1 Respiratory Diseases Unit, Department of Medical and Surgical Sciences, University Hospital of Modena and Center for Rare Lung Diseases, University of Modena Reggio Emilia, Modena, Italy; 2 Pathologic Anatomy Unit, Azienda USL Ravenna, Ravenna, Rimini Italy; 3 Respiratory Diseases Unit, University of Padua, Padua, Italy; 4 Respiratory Unit, University of Milano Bicocca, S. Gerardo Hospital, Monza, Italy; 5 Clinical and Experimental Medicine PhD Program, University of Modena Reggio Emilia, Modena, Italy; 6 Dipartimento di Scienze Chirurgiche e Diagnostiche Integrate, Università degli Studi di Genova, Genoa, Italy; 7 Ospedale Policlinico San Martino, IRCCS per l’Oncologia e le Neuroscienze, Genoa, Italy; 8 Intensive Care Unit Department, University Hospital Brussels (UZB), Jette, Belgium; 9 Faculty of Medicine and Pharmacy, Vrije Universiteit Brussel (VUB), Brussels, Belgium

**Keywords:** Interstitial lung diseases, Acute respiratory distress syndrome, Respiratory failure, Mechanical ventilation, Ventilator-induced lung injury

## Abstract

Protective ventilation is the cornerstone of treatment of patients with the acute respiratory distress syndrome (ARDS); however, no studies have yet established the best ventilatory strategy to adopt when patients with acute exacerbation of interstitial lung disease (AE-ILD) are admitted to the intensive care unit. Due to the severe impairment of the respiratory mechanics, the fibrotic lung is at high risk of developing ventilator-induced lung injury, regardless of the lung fibrosis etiology. The purpose of this review is to analyze the effects of mechanical ventilation in AE-ILD and to increase the knowledge on the characteristics of fibrotic lung during artificial ventilation, introducing the concept of “squishy ball lung”. The role of positive end-expiratory pressure is discussed, proposing a “lung resting strategy” as opposed to the “open lung approach”. The review also discusses the practical management of AE-ILD patients discussing illustrative clinical cases.

## Background

Interstitial lung diseases (ILD) represent a group of heterogeneous clinical conditions of both idiopathic and secondary nature, characterized by the coexistence of various degrees of inflammation and lung fibrosis [1, 2]. Many patients with ILD can develop an acute exacerbation in the course of the disease (AE-ILD), and often require ICU hospitalization and mechanical ventilation (MV). Idiopathic pulmonary fibrosis (IPF) is the most common and severe form of idiopathic ILD, often worsened by acute exacerbation episodes (AE-IPF). During these dramatic events, the typical usual interstitial pneumonia pattern (UIP)—the radiologic and histologic hallmark of IPF is overlapped with diffuse alveolar damage (DAD), sharing similarities with the acute respiratory distress syndrome (ARDS) [3]. Little is known about the outcome of latter patients receiving MV, and the influence of the extent of lung fibrosis component on ventilator management [4].

The purposes of this viewpoint paper are: (1) to describe the mechanical characteristics of the fibrotic lung during MV, introducing the concept of *“squishy ball lung”* and (2) to discuss the impact of MV in ICU patients with acute exacerbations of ILD.

## Specific pathophysiology

Independent of the underlying condition, the fibrotic lung has particular structural, biochemical and anatomical alterations resulting in profound changes in the mechanics of breathing.

### The extracellular matrix in the fibrotic lung

**Fig. 1:**
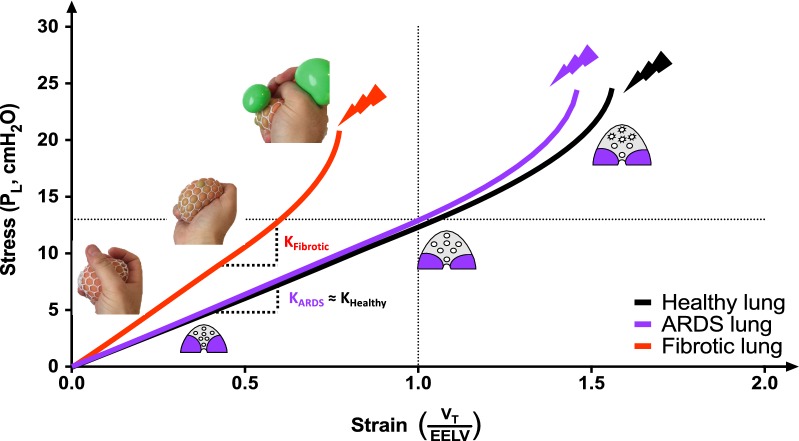
Relationship between stress and strain in healthy, ARDS and fibrotic lungs. The specific elastance (*K*) is the slope of the curve in its linear portion. Although ARDS lungs are characterized by low compliance, its elastic properties follow those of healthy lungs provided that the deformation induced by tidal ventilation is normalized to the end-expiratory lung volume. In ARDS, the “baby lung” (gray area) inflates until a certain level where hyperinflation occurs and the linearity of the stress–strain relation is lost, approaching the breakdown limit of the extracellular matrix constituents (lightning). In fibrotic lungs, the specific elastance is higher thus the stress–strain curve is steeper. During inflation, the healthy regions protrude through the fibrotic walls, as illustrated by the hand progressively squeezing the “squishy ball”. Compared to ARDS, the breakdown is reached at lower stress and lower strain. *ARDS* acute respiratory distress syndrome, *V*_T_ tidal volume, *EELV* end-expiratory lung volume, *P*_L_ transpulmonary pressure

The extracellular matrix (ECM) consists of a complex network of protein structures (collagen, fibronectin, elastin, glycoproteins and proteoglycans), which play a crucial role in determining the mechanical stability and elastic recoil of the lung. The ECM is a dynamic structure, constantly remodeled by enzymatic processes. In the fibrotic lung, there is a dysregulation of this remodeling process, with imbalance between protein secretion and degradation, with an increase in the deposition of collagen, elastin, proteoglycans and fibronectin [5]. Considering that the main stress-bearing constituents of lung tissue are collagen and elastin fibers, their quantitative and architectural modification can influence the elastic recoil of the lung. Elastin and collagen differ significantly in their mechanical properties. In fact, elastin is responsible for elasticity, especially at low stress levels, and can be stretched by more than 250% of its original length before breaking, while collagen is more rigid and significantly less stretchable being extendable only by 1–2% compared to the initial length [6]. Collagen fibers, which in the resting position are folded, are stretched only at high pulmonary volumes, close to the total lung capacity, and act as a blocking system determining the limitation of distention of the lung, and the origin of the curvilinear stress–strain relationship [7, 8] (Fig. 1). Therefore, elastin fibers are the main determinants of the maximum pulmonary volume that can be reached during inflation, beyond which there is a risk of barotrauma and volutrauma due to the breakdown of collagen fibers. This concept can be applied not only to the entire lung, but also to the different lung regions that have their maximum total regional capacity [7]. This is particularly relevant in the fibrotic lung, where the composition of the ECM has a high regional heterogeneity. In IPF, collagen fibers accumulate around myofibroblasts in fibroblastic foci, stiffening the corresponding regions [9].

### Histopathological characteristics of the fibrotic lung

Several histopathological patterns can characterize the lung during AE-ILD; among these, the most severe and common manifestation is the coexistence of DAD overlapped to a UIP pattern. The histopathological hallmarks of the UIP pattern are spatial heterogeneity, temporal heterogeneity with fibroblastic foci and micro-honeycombing. Spatial heterogeneity is defined as the presence of areas of normal tissue interposed to areas with fibrotic alterations. Temporal heterogeneity is the concomitant presence of areas with only slight modifications of the ECM structure and proliferative fibroblast and myofibroblasts aggregates, adjacent to areas of intense fibrosis composed of dense acellular collagen, indicating different coexisting stages of the disease. Honeycomb lesions are areas consisting of dilated air spaces with anelastic walls of epithelium-coated fibrous tissue [10]. Given these premises, it is clear how the mechanical properties of the fibrotic lung must reflect this histological heterogeneity.

### Mechanical properties of the fibrotic lung

The lung is commonly modeled as an elastic body characterized by minor distortions during inflation. In the non-fibrotic lung, the properties of the parenchyma can be described using two independent elastic modules, which are a function of the transpulmonary pressure (*P*_L_). The bulk modulus describes the lung behavior during uniform expansion, while the shear modulus (*G*) describes the non-uniform distortion behavior [11]. The shear modulus modifies approximately linearly as a function of transpulmonary pressure according to the following equation:

1 $$ \begin{array}{*{20}c} {G = \alpha \, \cdot P_{\text{L}}}, \\ \end{array} $$

where *α* represents the constant of proportionality that is variable according to mammal species.

The relationship between stress and strain is determined by the relationship:

2 $$ \begin{array}{*{20}c} {\text{Stress} = Y\, \cdot \text{Strain}}, \\ \end{array} $$

where the proportionality constant *Y* is the Young’s modulus. Stress is the equal and opposite force that develops in an elastic material when an external force is applied, namely the transpulmonary pressure (*P*_L_), while strain is the resulting deformation compared from the resting position, thus the ratio of the tidal volume (*V*_T_) to the end-expiratory (resting) lung volume (EELV). Equation 2 can thus be rewritten as follows:

3 $$ \begin{array}{*{20}c} {P_{L} = K\, \cdot \frac{{V_{T} }}{{\text{EELV}}}}, \\ \end{array} $$

where *K* corresponds to the specific elastance (Fig. 1), a coefficient describing the elastic properties of the lung whose value in healthy humans is around 13.5 cmH_2_O [12]. It can be interpreted as the *P*_L_ resulting in lung volume doubling compared to the EELV. When the *P*_L_ results in a lung volume above the total lung capacity, stretching of the collagen fibers occurs, causing VILI. Therefore, stress and strain are major determinants of VILI, respectively, involved in barotrauma and volutrauma.

This simple model is not applicable in presence of severe distortion of the pulmonary parenchyma, where *P*_L_ is no longer a function of linear elasticity modules, such as occurs in the fibrotic lung where anatomical inhomogeneities result in an anisotropic behavior: the application of *P*_L_ in a lung with a patchwork of mechanical–elastic properties has unpredictable consequences on the stress–strain coupling of the various areas of the lung, with high parenchymal distortion during insufflation and consequent increased risk of VILI. In fibrotic lungs, the high retraction forces due to the increased parenchymal rigidity might translate into reduced overall strain. Nevertheless, given the parenchymal heterogeneity, the lung zones without fibrosis might be subjected to intense deformation. In fact, in presence of relevant inhomogeneities the macroscopic lung mechanics parameters do not necessarily reflect what happens at the micro-scale, where inhomogeneities act as local stress raisers and increase the local *P*_L_ [13].

### The squishy ball lung theory

**Fig. 2:**
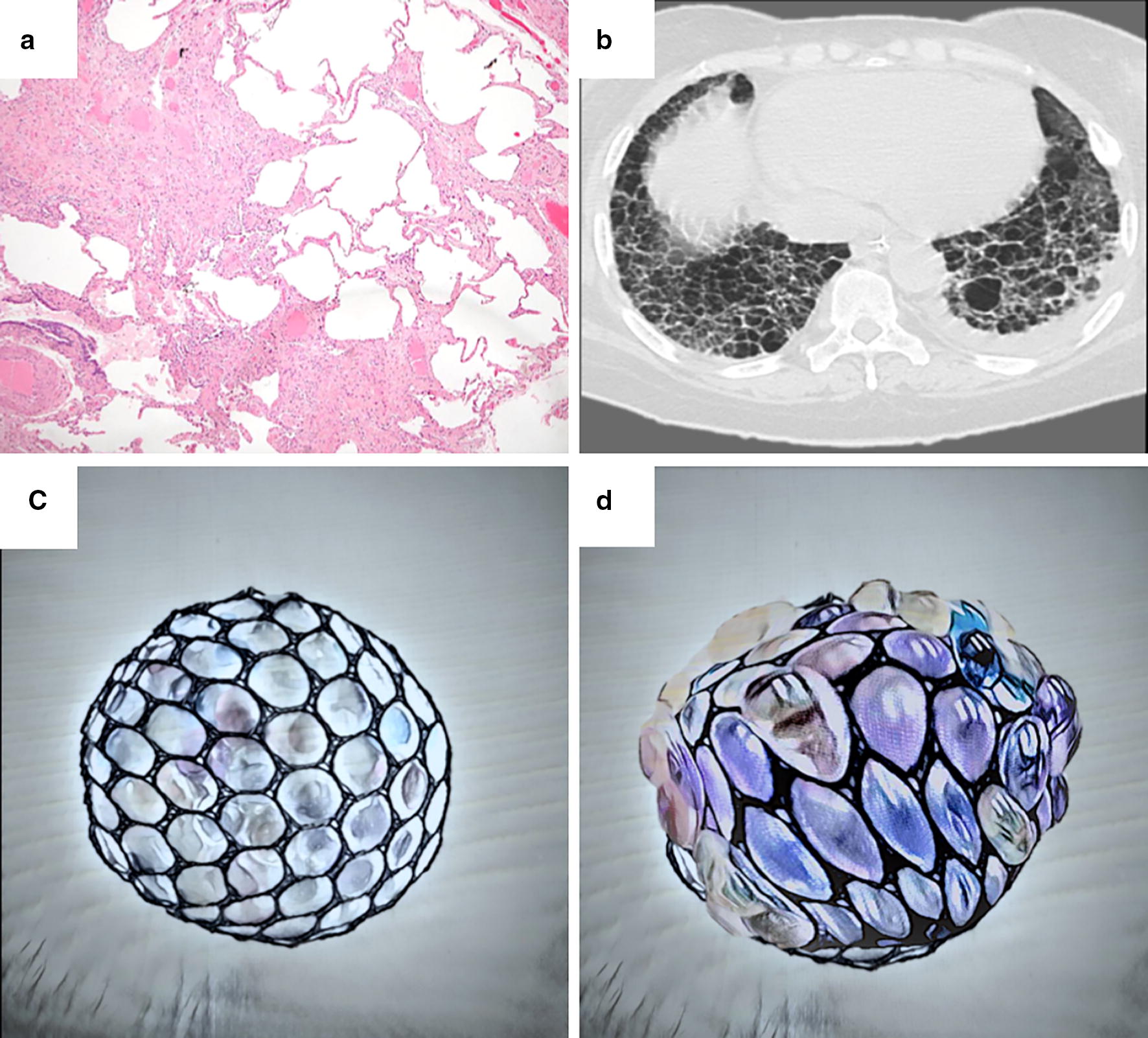
**a** Histological evidence of spatial heterogeneity with relatively spared alveolar spaces surrounded by patchy areas of fibrosis with multiple fibroblastic foci in a patient with IPF. **b** CT appearance of UIP pattern in a patient with IPF. **c** Graphical appearance of a “squishy ball” depicting the elastic features of fibrotic lung in resting position. **d** Squishy ball subjected to the application of an internal pressure: the increase of the pressure inside the object causes throttling of the elastic part of the body through the inelastic net that wraps the ball determining a mechanical disadvantage during the expansion

In fibrotic lungs, the effect of PEEP can determine the protrusion of the most distensible lung areas through dense anelastic fibrotic tissue circles, causing increased rigidity and facilitating tissue breakdown. The effect that is determined in some areas of the lung is similar to that shown in stress balls called ‘squishy balls’ (Figs. 1 and 2). When the squishy ball is compressed, the increase of the pressure inside the object causes throttling of the elastic part of the body through the inelastic net that wraps the ball. The result is the formation of vesicles that protrude outside the net mesh, until reaching the elastic limit. The “squishy ball effect” in some areas of the lung may be the cause of mechanical disadvantages achieved using high P_airway_ and *P*_L_ in the lungs with fibrosis and could confirm the role of static strain in generating VILI. Moreover, when the most recruitable areas are subject to high *P*_L_, the subsequent overinflation is exacerbated by the mechanical geometry of the fibrotic lung as the anelastic areas act as stress raisers.

### Clinical implications

The mentioned pathophysiological and histological characteristics of AE-ILD have implications for the application of MV, titration of PEEP and respiratory monitoring.

### Mechanical ventilation and clinical outcome in patients with AE-ILD

Low tidal volume protective MV is widely recognized as the cornerstone in the treatment of ARDS patients, while in patients with AE-ILD admitted to the ICU, studies have not established yet the best ventilatory strategy. As illustrated above, patients receiving MV for AE-IPF have severe alterations in respiratory mechanics with an increase in the elastance of the respiratory system, mainly due to an abnormal lung elastance while chest wall elastance may be normal (Table 1) [14]. Based on the concepts derived from physiologic studies, experts recommend keeping the static *P*_L_ at end-inspiration below 15–20 cmH_2_O in homogeneous and below 10–12 cmH_2_O in inhomogeneous lung parenchyma, such as in ARDS.

**Table 1:** Lung mechanical properties of three patients experiencing acute exacerbation of interstitial lung disease (AE-ILD)

Measurement	Patient 1 (CHP)		Patient 2 (IPF)		Patient 3 (CHP)	
PEEP titration strategy	Lung resting strategy	Open lung approach	Lung resting strategy	Open lung approach	Lung resting strategy	Open lung approach
Set PEEP (cmH_2_O)	4	12	4	12	4	12
Driving pressure (cmH_2_O)	17.0	18.0	14.5	18.0	12.0	16.0
Transpulmonary pressure (cmH_2_O)						
End-inspiratory	14.0	16.7	9.9	16.0	10.0	13.9
End-expiratory	− 2.2	0.2	− 4.0	0.3	− 1.0	1.6
Driving pressure	16.2	16.5	14.0	16.3	11.0	12.3
Elastance (cmH_2_O/L)						
Respiratory system	44.6	51.6	34	47	40	43
Pulmonary	42.5	47.0	33.0	45.0	35.0	37.9
Chest wall	2.1	4.6	1.0	2.0	5.0	5.8
Blood arterial PaO_2_/FiO_2_ (mmHg)	92	78	113	110	85	79

Patient 1 and 3 presented chronic hypersensitivity pneumonitis (CHF) while patient 2 presented idiopathic pulmonary fibrosis (IPF) In each patient, two PEEP setting strategies were tested: a *“lung resting strategy”* aimed at minimizing PEEP while maintaining sufficient oxygenation (SpO_2_ > 88–92%) and an “*open lung approach”* titrating PEEP aiming at avoiding negative end-expiratory transpulmonary pressure. The negative end-expiratory transpulmonary pressure values achieved at 4 cmH_2_O PEEP suggest that low levels of PEEP do not prevent tidal alveolar de-recruitment. Nevertheless, higher levels of PEEP determined mild-to-critical increase in lung elastance and non-clinically relevant worsening of gas exchange

*PEEP* positive end-expiratory pressure

While several studies show that in IPF patients the need for MV is associated with high mortality, little is known about the prognostic impact of MV in ILD other than IPF [15, 16]. In a recent cohort study in patients with ILD of different etiology hospitalized for acute respiratory failure, survival at 60 months was comparable in IPF and non-IPF patients and ICU admission and the use of MV were the only independent predictors of in-hospital death [17]. Nonetheless, when patients with AE-ILD of different etiology receive MV, the presence of pulmonary hypertension and the evidence of diffuse fibrosis on CT scan are associated with worse prognosis, while the radiologic extension of lung fibrosis is directly correlated with worse respiratory mechanics and increased mortality [18].

Interestingly, in a case series of mechanically ventilated patients with interstitial pneumonia with autoimmune features, mortality was lower compared to patients with ARDS of known cause [19]. These data may sound surprising but can be linked to the peculiar radiological patterns reported in the series as none of the patients presented a UIP pattern on CT scan, while signs of inflammatory alveolar disease and ground-glass opacities were predominant. These observations suggest that the prognosis of patient with ILD in MV is related to the extension of the lung fibrosis and the presence a UIP pattern on CT scan rather than to the ILD etiology.

### Effects of PEEP in AE-ILD

In ARDS, lung protection is provided using low tidal volumes, low plateau transpulmonary and driving pressures, but also a positive end-expiratory pressure (PEEP) level sufficient to maintain oxygenation while preventing the opening–closing of alveolar units causing shear stress throughout the respiratory cycle [20]. Clinical trials in ARDS investigated the effect of an *open lung* strategy, namely involving the use of PEEP levels higher than those strictly required to maintain acceptable oxygenation [21], often in conjunction with recruitment maneuvers to maximize lung aeration [22]. Such studies have not been able to show clear advantages in terms of outcome compared to ventilation with lower PEEP levels. Furthermore, an aggressive recruitment strategy used in one study resulted even in increased mortality [23]. Some authors started to suggest that lung pressures, including PEEP, should be minimized to reduce VILI in patients with injured and non-injured lungs [24–26]; these concepts seem to be promising also for fibrotic lungs where susceptibility to VILI is particularly high.

Interestingly, in the patients with fibrotic lung and superimposed DAD, retrospective data showed an association between higher PEEP levels and mortality [16]. Compared to ARDS, physiology of MV in IPF patients is much less known [3], and it is unclear whether opening and closing of alveolar units during tidal breathing occurs, as what exactly the role of PEEP is on alveolar recruitment.

Monitoring *P*_L_ through esophageal pressure assessment [27] has been proposed to identify patients with regional alveolar collapse at the end of expiration, suggested by a negative end-expiratory *P*_L_. Physiologic studies confirmed that *P*_L_ estimated by esophageal manometry reflects the regional *P*_L_ of dependent lung areas where atelectasis predominate [28, 29]. Titrating PEEP to target a positive *P*_L_ at end-expiration maximizes lung recruitment and improves respiratory mechanics and oxygenation in ARDS [30], but did not improve survival in ARDS when compared to empirical high PEEP [31]. This particular technique is one of the methods proposed to achieve an “open lung approach”. However, despite decades of intense clinical research in ARDS, ventilatory strategies aimed at achieving an *‘open lung’* (open the lung and keep it open) with the use of PEEP failed to translate these findings in the clinical setting [32], and some author suggested the ‘lung rest’ (close the lung and keep it resting) strategy [24].

Despite the lack of physiological data in AE-ILD patients, it might be assumed that expiratory derecruitment occurs in parenchymal areas spared from fibrosis with preserved elasticity. Despite a possible role of incremental PEEP in the recruitment of these areas, the reported association between higher PEEP levels and mortality in AE-ILD [16], indicates a critical role of static strain in determining VILI in patients with fibrotic lungs, and might suggest that limiting airway pressures, including PEEP, could be preferable.

## Practical management tips

This paragraph illustrates practical aspects of the clinical management of patients with AE-ILD.

### Clinical pathway of patients with AE-ILD

Overall, AE-ILD has a poor prognosis and the choice to initiate MV or to admit the patient to the ICU can be challenging, particularly in patients with IPF [3]. In several clinical settings, ICU physicians tend to be reluctant to admit IPF patients if they are not already listed for transplant, considering invasive ventilation as a *bridge*-*to*-*transplant* therapy [16]. Nevertheless, evidence shows that also patients with ILD other than IPF may present with acute exacerbation during the natural course of the disease [33] requiring ICU admission and MV [15, 33, 34]. Autopsy studies show that the majority of patients who died from AE-ILD other than IPF often present with a DAD superimposed on a UIP pattern at the histologic examination, resembling what usually is found on biopsies of patients that died from AE-IPF [34, 35]. The presence of a UIP pattern on histology is strictly correlated with peculiar features on CT scan, namely radiological UIP pattern [33]. Therefore, intensivists should be able to promptly recognize the UIP pattern at the CT scan, as it is the main determinant of the *squishy*-*ball* behavior of the fibrotic lung subject to MV.

### How to identify the AE-ILD radiological pattern

The correct identification of UIP pattern at the CT scan can be useful in the clinical evaluation of patients with AE-ILD whose lung mechanical substrate is more prone to the development of VILI once subjected to MV with worst clinical outcomes. The ultimate guidelines on diagnosis of IPF defined the typical radiographic features of UIP pattern on CT. The radiologic hallmark of UIP is the presence of honeycombing, multiple layers of sub-pleural clustered cystic airspaces with thick, well-defined walls and typically consistent diameter (3–10 mm, but occasionally larger). A fine reticular pattern containing traction bronchiectasis ranging from subtle irregularity of the bronchial/bronchiolar wall to marked airway distortion and varicosity is another key feature of UIP pattern. The typical distribution of these abnormalities follows a cranio-caudal gradient with sub-pleural predominance [36]. Ground-glass opacifications as defined by hazy increased opacity of lung airspaces with substantial preservation of the bronchial and vascular margins on CT, represent the usual radiological appearance of inflammatory alveolar abnormalities, including DAD [37] but may be also present in patients with ILD [38]. When ground-glass opacifications result superimposed on a fine reticular pattern surrounded by traction bronchiectasis they should be referred to alveolar fibrosis and might identify a subgroup of patients at extremely high risk of VILI [39]. In summary, in the context of ILD of different etiology, the presence of a UIP pattern identifies a mechanical substrate more prone to VILI as a consequence of the *“squishy ball”* behavior. In this setting a lung resting approach might be preferable to prevent possible damages. In AE-ILD patients with ground-glass abnormalities in the absence of significant UIP pattern, mechanical behavior of the lung might be similar to ARDS.

## How to set mechanical ventilation

There is lack of specific evidence concerning MV settings in AE-ILD. Some of the recommendations can be derived from the evidence concerning ARDS, but several other aspects need to be elucidated in further research [3]. Advanced respiratory monitoring, including esophageal pressure where available, is important to identify those patients more prone to VILI [40].

Concerning tidal volume, we recommend targeting 6 ml/kg of predicted body weight, as established in ARDS [41]. In case of high driving and/or plateau pressures a further reduction could be considered [42], however this strategy in AE-ILD can lead to unacceptable hypercapnia. The respiratory rate should be set to avoid respiratory acidosis, tolerating hypercapnia if the arterial pH remains above 7.25. Attention should be paid to the presence of intrinsic PEEP, namely a careful inspection of the flow–time curve should be performed to ensure that the expiratory flow reaches zero at end-expiration.

The use of high PEEP levels does not seem appropriate, due to the peculiar characteristics of the fibrotic lung. We advocate the adoption of a *“lung resting strategy”*, tolerating moderate atelectasis titrating PEEP to the minimal values necessary to achieve minimal oxygenation, i.e., an arterial partial pressure of oxygen above 50–60 mmHg or a SpO_2_ above 88–90%. In patients in whom a DAD or ground-glass opacities at the CT predominate over the UIP pattern, higher PEEP levels might be considered, similarly to ARDS.

### Illustrative cases

**Fig. 3:**
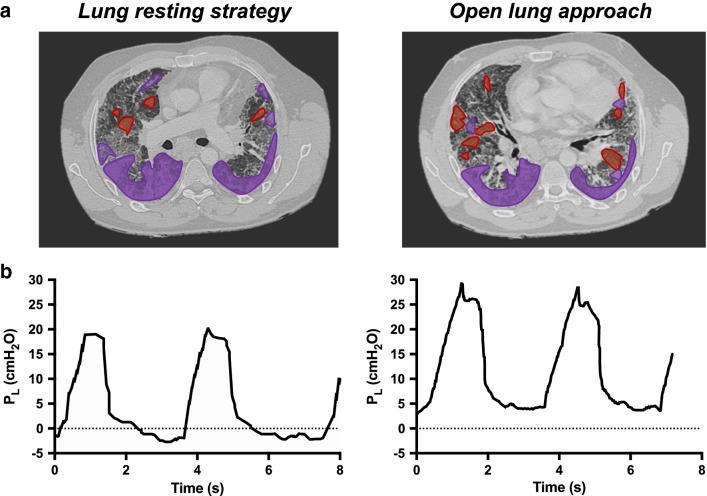
CT scan images and transpulmonary pressure monitoring of a representative patient with UIP pattern and superimposed ground-glass during an AE-ILD, with PEEP set according to a “lung resting strategy” (left, PEEP 4 cmH_2_O) or with an “open lung approach” titrated to achieve positive end-expiratory transpulmonary pressure (right, PEEP 12 cmH_2_O). End-inspiratory transpulmonary pressure values significantly rise when higher values of PEEP are applied. Purple areas represent lung collapse, opacities and fibrous regions. Red circles highlight areas of over-inflation. *AE* acute exacerbation, *ILD* interstitial lung disease, *UIP* usual interstitial pneumonia, *PEEP* positive end-expiratory pressure

We assessed retrospectively clinical data of three patients with AE-ILD of different etiology admitted to the Respiratory Intensive Care Unit of the University Hospital of Modena, Italy, from January 2016 to January 2018 to receive invasive controlled MV: one had IPF and two had chronic hypersensitivity pneumonitis (CHP). All patients presented a UIP pattern with superimposed ground-glass opacities on the CT scan (Fig. 3a). Patients were non-obese males (body mass index, mean ± standard deviation of 22.8 ± 2.3 kg/m^2^), aged 62.6 ± 9.1 (age at diagnosis 60 ± 8.5 years). All patients underwent transpulmonary pressure monitoring with esophageal manometry (Fig. 3b). Table 1 shows respiratory mechanics and gas exchange parameters of these patients, when PEEP was set according to a *“lung resting strategy”* (minimal PEEP level of 4 cmH_2_O) and after PEEP titration on an *“open lung approach”* aiming at achieving positive end-expiratory transpulmonary pressure values. In all patients, with minimal levels of PEEP aimed at achieving minimal acceptable oxygenation, end-expiratory *P*_L_ was negative. This suggests that even in the fibrotic lung with diffuse alveolar damage tidal de-recruitment of the dependent zones during the expiration might occur. Nevertheless, in these patients a PEEP titration strategy to maintain a positive end-expiratory *P*_L_ resulted in a significant mechanical disadvantage. In these patients, higher PEEP levels lead to an increase in the driving pressure, lung elastance and end-inspiration *P*_L_ values. This suggest that PEEP is able to counteract alveolar recruitment–derecruitment, but at the price of a remarkable lung parenchymal stress. The mechanical disadvantages determined by high PEEP, suggests that in the fibrotic lung with diffuse alveolar damage, the static strain might play a relevant role.

## Conclusions

The management of the patient with lung fibrosis in the ICU is a challenge for the intensivist. The lack of studies defining the mechanical ventilation strategy, and the different underlying etiologies, make it difficult to decide which patient can benefit from ICU admission and MV. The few data that are available show that the prognosis of patients with non-IPF pulmonary fibrosis subjected to MV is dependent on the degree of extensive fibrosis present on CT scan, rather than the underlying etiology. The architecture of the fibrotic lung makes it particularly fragile when subjected to high PEEP. The presence of conserved lung areas, next to areas of dense anelastic fibrosis, does not prevent the phenomenon of alveolar recruitment–derecruitment during tidal volume. The use of high PEEP to keep alveolar units opened during expiration exposes the lung at risk of injury by forming “squishy ball” lung areas that aggravate the end-inspiratory transpulmonary pressure effects. Pending further studies to define the optimal strategy to ventilate these lungs, we herein suggest using a *“lung resting strategy”*, as opposed *to “open lung approach”* in patients affected by pulmonary fibrosis and UIP pattern under MV, regardless of the underlying etiology.

## Data Availability

Not applicable.

## Abbreviations

AE: Acute exacerbation
IPF: Idiopathic pulmonary fibrosis
ILD: Interstitial lung diseases
UIP: Usual interstitial pneumonia
DAD: Diffuse alveolar damage
EELV: End-expiratory lung volume
ICU: Intensive Care Unit
PEEP: Positive end-expiratory pressure
MV: Mechanical ventilation
*V*_T_: Tidal volume
ARDS: Acute respiratory distress syndrome
VILI: Ventilator-induced lung injury
ARF: Acute respiratory failure
CHP: Chronic hypersensitivity pneumonitis
CT: Computed tomography
ECM: Extracellular matrix
